# Characterization and phylogenetic relationships analysis of the complete chloroplast genome of *Carya ovata*

**DOI:** 10.1080/23802359.2020.1797584

**Published:** 2020-07-30

**Authors:** Mengyang Xu, Yunzhou Lyu, Zhongren Guo, Zhenghai Mo

**Affiliations:** a Institute of Botany, Jiangsu Province and Chinese Academy of Sciences, Nanjing, China; b Jiangsu Academy of Forestry, Nanjing, China; c The Jiangsu Provincial Platform for Conservation and Utilization of Agricultural Germplasm, Nanjing, China

**Keywords:** *Carya ovata*, chloroplast genome, phylogenetic analysis

## Abstract

*Carya ovata* is a slow-growing, long-lived deciduous species that belongs to section *Carya* of genus *Carya*. In this study, we *de novo* assembled the complete chloroplast genome of *C*. *ovata*, and analyzed its phylogenetic relationship. The circular genome was 160,765 bp in length, comprising a large single-copy region (89,975 bp), a small single-copy region (18,788 bp), and a pair of inverted repeat regions (26,001 bp each). The chloroplast genome was predicted to contain 131 genes, including 83 protein-coding genes, 40 transfer RNA (tRNA) genes, and 8 ribosomal RNA (rRNA) genes. Overall, the GC content of the chloroplast genome was 36.16%. Phylogenetic analysis suggested that *C*. *ovata* was closely related to *C. illinoinensis*, a representative of section *Apocarya* within the genus *Carya*.

*Carya ovata*, commonly known as shagbark hickory, is a member of family Juglandaceae (Thompson and Grauke 1991). This species is distinguished by its conspicuous and persistent dark outer bud scales over the winter buds. Previously, the phylogenetic position of *C*. *ovata* was mainly determined according to its geographical distribution and morphology (Manos and Stone 2001). An application of comparative genomic date to illustrate its phylogenetic relationships is still lacking. Chloroplast genome of angiosperms is maternal inheritance and displays a relatively slow rate of evolution compared to nuclear genomes (Daniell et al. 2016; Liu et al. 2016; Mo et al. 2020). Therefore, the polymorphic regions in chloroplast genomes are commonly used for phylogenetic analysis (Chen et al. 2018). In this study, we *de no* assembled the *C*. *ovata* chloroplast genome and analyzed its phylogenetic position on the molecular level.

Fresh and healthy leaves of *C*. *ovata* was collected from experimental farm of Jiangsu Academy of Forestry (Nanjing, China 118°45′57.30″E, 31°51′27.94″N), and stored at −80 °C until further use. Its voucher specimens are preserved in the Herbarium of Jiangsu Academy Forestry (JAF: Lyu20200512-3). Total genomic DNA of *C*. *ovata* was isolated by using Tiangen Plant Genomic DNA Kit (Tiangen Biotech Co., Beijing, China). The extracted DNA was broken into small fragments to be used for paired end library construction. The genomic library was sequenced on an Illumina Hiseq 4000 platform. After the completion of sequencing, raw reads were generated and were further processed to obtain clean reads by using the Trimmomatic (v0.32) software. The annotated chloroplast genome of *C*. *illinoinensis* was downloaded from GenBank (accession number: MH909600), and was used as reference for *C*. *ovata* chloroplast genome assembly through the NOVOPlasty software. Chloroplast genome annotation was performed by using the online annotation program DOGMA, and manually checked by Blast search. The annotated chloroplast genome was deposited in GenBank (Accession number: MT701613).

The *C*. *ovata* chloroplast genome was 160,765 bp in length, including two copies of inverted repeats (IRa and IRb) of 26,001 bp that were separated by a large single-copy (LSC) region of 89,975 bp and a small single-copy (SSC) region of 18,788 bp. The *C*. *ovata* chloroplast genome was predicted to contain 131 genes: 83 protein-coding genes, 40 tRNA genes, and 8 rRNA genes, 18 of which were duplicated in the IR regions, giving 113 unique genes totally. The chloroplast genome consisted of 48.76% coding regions and 51.24% non-coding regions (including both intergenic spaces and introns). Overall, the GC content contained in the chloroplast genome is 36.16%.

Phylogenetic analysis was performed based on the complete chloroplast genome sequences of 21 species: 14 from Juglandaceae, 3 from Betulaceae, 3 from fagaceae, and 1 outgroup (*Populus richocarpa*). We firstly used MAFFT to align the multiple sequences, and then applied IQ-tree to construct phylogenetic tree with the maximum-likelihood method (Katoh and Standley 2013; Nguyen et al. 2015) (Figure 1). The genus *Carya* is currently divided into three section: sect. *Carya*, sect. *Apocarya*, and sect. *Sinocarya* (Manos and Stone 2001). *Carya ovata* and *C. illinoinensis* are representatives of sect. *Carya* and sect. *Apocarya*, respectively. *Carya cathayensis* and *C. kweichowensis* are the representative species of sect. *Sinocarya*. Our phylogenetic analysis indicated that *C*. *ovata* was closely related to the species of sect. *Apocarya*, represented by *C. illinoinensis*.

**Figure 1.**
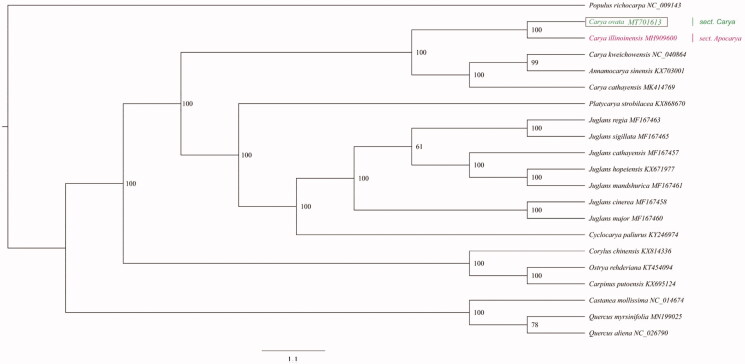
Phylogenetic tree construction using maximum likelihood (ML) based on 21 complete chloroplast genome sequences. The bootstrap support values were shown at the branches.

## Data Availability

The data that support the findings of this study are openly available in NCBI at http://www.ncbi.nlm.nih.gov/, reference number MT701613.
